# A pathway activity-based proteomic classifier stratifies prostate tumors into two subtypes

**DOI:** 10.1186/s12014-023-09441-w

**Published:** 2023-11-11

**Authors:** Rui Sun, Lingling Tan, Xuan Ding, Jun A, Zhangzhi Xue, Xue Cai, Sainan Li, Tiannan Guo

**Affiliations:** 1 Westlake Center for Intelligent Proteomics, Westlake Laboratory of Life Sciences and Biomedicine, Hangzhou, Zhejiang China; 2 Key Laboratory of Structural Biology of Zhejiang Province, School of Life Sciences, Westlake University, Hangzhou, 310024 China; 3 Institute of Basic Medical Sciences, Westlake Institute for Advanced Study, Hangzhou, 310024 China; 4 Westlake Omics (Hangzhou) Biotechnology Co., Ltd., Hangzhou, 310024 China

**Keywords:** Prostate cancer, PulseDIA, Proteomic pathway-based classifier, BCR-free survival

## Abstract

Prostate cancer (PCa) is the second most common cancer in males worldwide. The risk stratification of PCa is mainly based on morphological examination. Here we analyzed the proteome of 667 tumor samples from 487 Chinese PCa patients and characterized 9576 protein groups by PulseDIA mass spectrometry. Then we developed a pathway activity-based classifier concerning 13 proteins from seven pathways, and dichotomized the PCa patients into two subtypes, namely PPS1 and PPS2. PPS1 is featured with enhanced innate immunity, while PPS2 with suppressed innate immunity. This classifier exhibited a correlation with PCa progression in our cohort and was further validated by two published transcriptome datasets. Notably, PPS2 was significantly correlated with poor biochemical recurrence (BCR)/metastasis-free survival (log-rank P-value < 0.05). The PPS2 was also featured with cell proliferation activation. Together, our study presents a novel pathway activity-based stratification scheme for PCa.

**Supplementary Information:**

The online version contains supplementary material available at 10.1186/s12014-023-09441-w.

## Introduction

Prostate cancer (PCa) is the second most common malignancy with the fifth-highest mortality among the male population worldwide [1]. The difficulty of studying prostate cancer is the scarcity of survival data. The Gleason scoring system/International Society of Urological Pathology (ISUP) grade is widely used to predict survival outcomes [2]. PCa generally exhibits a more favorable prognosis compared to other malignant tumors, with 5-year PCa-specific mortality-free survival rates exceeding 90% for 1–4 ISUP grades [3]. However, accurately classifying ISUP grades presents challenges and is inherently subjective, leading to inter- or intra-pathologist variability [4, 5]. Since this variability can lead to both under-grading or over-grading of Pca [5–7], more precise diagnostic tests are still in urgent needs.

Genomic and transcriptomic studies have proposed PCa classifications, based on genomic alterations such as *SPOP*, *FOXA1*, *IDH1,* and ETS fusion [8–11]. However, their prognosis values remain unclear. Recently, a multi-omic study revealed that the high genomic heterogeneity could be buffered at the proteomic level [12]. Ankit *et.al* found that the proteomic features of prognostic biomarkers are superior to the genomic and transcriptomic features in 76 PCa patients [13]. This finding is also supported by several other cancer studies [13–16]. Thus, a comprehensive proteomic analysis of PCa is urgently needed. Also, rather than investigating a single gene or protein, a proteomic pathway activity-based analysis provides a deeper understanding of the molecular mechanisms of PCa. Furthermore, the integration of protein complexes, pathways, and networks improves the phenotype prediction compared with a single protein as shown in COVID-19 [17]. However, no study has developed proteomic pathway-based clinical classifiers to facilitate the diagnosis or prognosis of PCa patients.

Data-independent acquisition mass spectrometry (DIA-MS)-based proteomics analysis has been widely used for the exploration of novel biomarkers and therapeutic targets [18]. PulseDIA, a combination of gas phase fractionation and DIA, can further improve the depth and robustness of proteomics compared with DIA [19]. Here, we profiled the proteome of 487 Chinese PCa patients using PulseDIA to explore new means of performing risk prediction for PCa and understanding the molecular mechanism of PCa development. The main objective of this study is to elucidate the molecular alterations associated with prostate tumor survival. Indeed, our subtype cannot be directly applied in clinic at the moment; however, we anticipate that it has the potential to be implemented as an independent and complementary test for the Gleason scoring/ISUP grading.

## Results and discussions

### Quantitative proteomic analysis

**Fig. 1:**
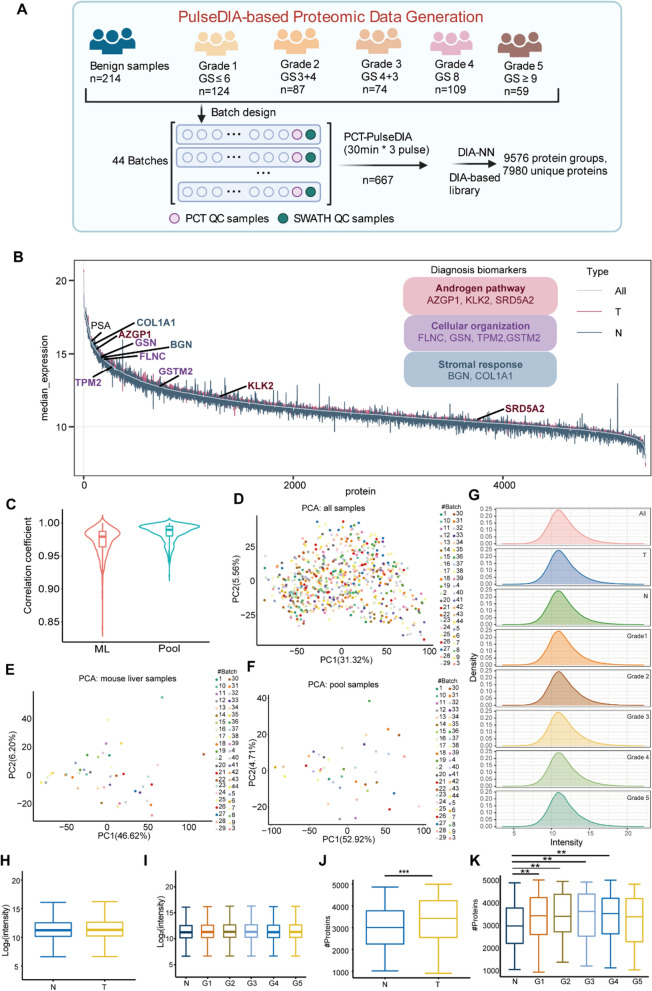
**A** Study design of the molecular classification for PCa. A total of 453 FFPE prostate tissue samples from 5 different ISUP grades and 214 benign samples were used for proteomic analysis. **B** The median protein abundance of each protein across all samples. **C** The Pearson correlation distribution of the quality control samples including the mouse liver (ML) samples and pool PCa samples. **D**–**F** PCA plots for 44 batches, including all samples (**D**), ML samples (**E**), and pool PCa samples (**F**). **G** Density plot for each PCa type. **H**–**I** Protein quantification between different ISUP grades (**H**) and sample types (**I**). **J**–**K** The number of proteins identified in the tumor and adjacent benign samples (**J**), and in the different ISUP grades (**K**). P-value: * < 0.05; ** < 0.01; *** < 0.001. T, tumor samples; N, adjacent benign samples

We collected 667 formalin-fixed, paraffin-embedded (FFPE) prostate tissue samples from 487 Chinese patients, including 182 paired tumor and adjacent benign samples, 271 unpaired tumor samples, and 32 unpaired adjacent benign samples (Additional file 1: Table S1A). The tumor samples were graded using the International Society of Urological Pathology (ISUP) standard [2], ranging from grade 1 (GS ≤ 6) to 5 (GS ≥ 9) (Fig. 1A). We identified 9576 protein groups (corresponding to 7980 unique proteins) by pressure cycle technology (PCT) coupled with PulseDIA [19] on a TripleTOF mass spectrometer (Fig. 1A). After removing proteins absent in more than 80% of the samples, 5360 protein groups and 4413 unique proteins were quantified (Additional file 1: Table S1B). Known PCa biomarkers were detected, including PSA (Fig. 1B). Our data also included nine proteins from a 12-gene tissue-based diagnostic kit for PCa (Oncotype DX20) (Fig. 1B). These proteins are associated with the androgen pathway, cellular organization, proliferation, and stromal response [20]. The median correlation coefficients of the quality control samples (mouse liver samples for PCT quality control and pool samples for LC–MS/MS control) were over 0.95 (Fig. 1C). The random distribution of all samples (Fig. 1D), mouse liver samples (Fig. 1E), and pool samples (Fig. 1F) showed few batch effects among different batches in the processes of sample preparation and proteomics data acquisition, respectively. All of the quality control analyses suggested that the MS data was of high quality. The protein intensity distributions among different sample types (Fig. 1G) were similar, including tumor and normal samples (Fig. 1H), and different ISUP grades (Fig. 1I). However, more proteins were identified in the tumor samples (Fig. 1J) and the higher GS groups (Fig. 1K) on average, which was consistent with the previous study [21].

### Proteomic pathway-based stratification for PCa

**Fig. 2:**
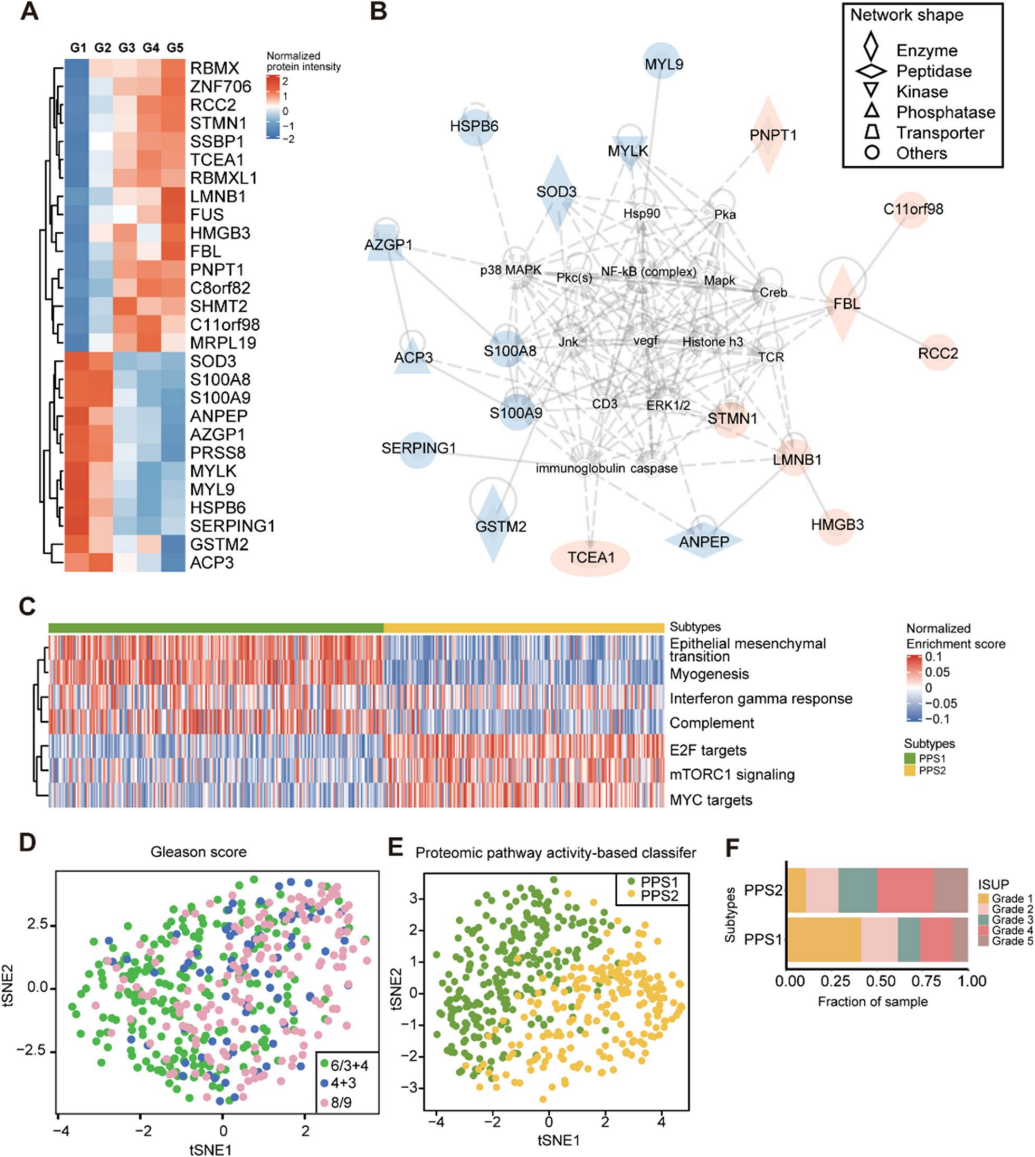
Proteomic pathway-based classifier.** A** Heatmap of 28 overlapping proteins that were significantly differentially expressed between tumor and adjacent benign samples (B-H adjusted P-value < 0.05, fold change > 2 or < 0.5), and 4 clusters (cluster 2, 5, 8, 10 in Additional file 2: Figure S1B) from mFuzz analysis (one-way ANOVA, B-H adjusted P-value < 0.05). Proteins that exhibit an increasing trend with ISUP grades are indicated by the color red, while those with a decreasing trend are represented by blue. Proteins that were not detected in our dataset are denoted by gray. Different shapes reflects the diverse biological functions of the proteins. **B** The protein–protein interaction network of the 28 proteins from STRING. **C** An unsupervised classifier based on proteomic pathways. **D**–**E** The t-SNE shows the distribution of all tumor samples using ISUP standard and the pathway-based classifier. The classifier was based on the selected 13 proteins shown in Fig. 2A. **F** The overlay of proteomic pathway-based subtypes using the ISUP classification standard for PCa

We focused on the pathways that are most significantly affected in PCa. Firstly, we identified 733 differentially expressed proteins (DEPs) between the tumor and benign groups (Additional file 1: Table S2A), which were mainly enriched in EIF2 signaling, amino acid metabolism, oxidative phosphorylation, and splicing associated pathways (Additional file 2: Figure S1A). In our analysis of tumor samples across the five ISUP grades, we utilized ANOVA (Additional file 1: Table S2B) to identify 348 DEPs. These DEPs were then classified into ten clusters using the Mfuzz (version 2.48.0) package [22] (Additional file 2: Figure S1B). To explore the trends in these DEPs across different grades, we selected four clusters. Protein clusters 8 and 10 demonstrated a consistent increase from grades 1 to 5, whereas protein clusters 2 and 5 displayed a decreasing trend (Additional file 2: Figure S1B). We found a total of 28 DEPs (Fig. 2A) that overlapped from the previously described two comparisons: between tumor and benign groups, and among five grades (clusters 2, 5, 8, 10). Some of them were enriched in the renal and urological disease associated network (Fig. 2B). Among them, STMN1 [23] and HMGB3 [24] can promote the proliferation and metastasis of PCa tumor cells. FBL [25] and RBMX/RBMXL1 [26] all participate in RNA splicing and translation, which have been reported to be highly expressed in PCa and regulated by MYC. SHMT2 catalyzes serine decomposition to regulate metabolic reprogramming by the STAT3/SHMT2/PKM2 pathway [27]. SOD3 [28], PRSS8 [29], and GSTM2 [30] act as oncogenes in PCa, while downregulation of S100A8 [31], S100A9 [31], and MYL9 [32] is associated with a poor prognosis in PCa.

These 28 DEPs were enriched in eight dysregulated pathways (Additional file 1: Table S3A, B), including epithelial mesenchymal transition (EMT), myogenesis, interferon-gamma response, complement, G2M checkpoint, E2F targets, mTORC1 signaling, and MYC targets (Fig. 2C). However, only 13 proteins appeared in these eight pathways. Specifically, the pathways G2M checkpoint and E2F target only showed enrichment for two identical proteins. The activation of the E2F signaling pathway has been positively linked to androgen-dependent PCa metastasis [33]. Thus, we have preserved the E2F target pathway, and the subsequent analysis was conducted based on a classifier using seven pathways and 13 proteins. Compared to previous genomic and proteomic studies on PCa [8–12, 21, 34–37], our study analyzed the largest patient cohort and developed a pathway-based classifier that is associated with prognosis. The seven pathways involved have been sporadically reported to be linked with PCa. Among these, EMT, myogenesis, and inflammation-related pathways have been associated with a poor prognosis in PCa [37]. MYC has also been associated with the malignancy of PCa, while promoting TMPRSS2-ERG fusion [38]. The pathway enrichment scores of the seven pathways were estimated in each sample using gene set variation analysis (GSVA). According to the score, the 478 tumor samples were optimally classified into two groups (Additional file 2: Figure S2), namely PPS1 and PPS2. Although 13 DEPs were insufficient to differentiate between ISUP grades (Fig. 2D), our proteomic pathway activity-based classifier was able to effectively categorize PCa patients into two distinct groups (Fig. 2E). Our analysis revealed that PPS1 had a higher proportion of low-risk PCa patients (ISUP grades 1–3) and a lower proportion of high-risk patients (ISUP grades 4–5). (Fig. 2F). PPS1 is characterized by innate immune activation, while MYC targets, and mTORC1 signaling are activated in PPS2 (Fig. 2C). Our data suggested that innate immunity might be activated in low-grade patients, while cell proliferation associated signaling pathways were activated in high-grade PCa patients.

### Innate immune suppression and cell proliferation activation predicted short BCR-free survival in PCa patients

**Fig. 3:**
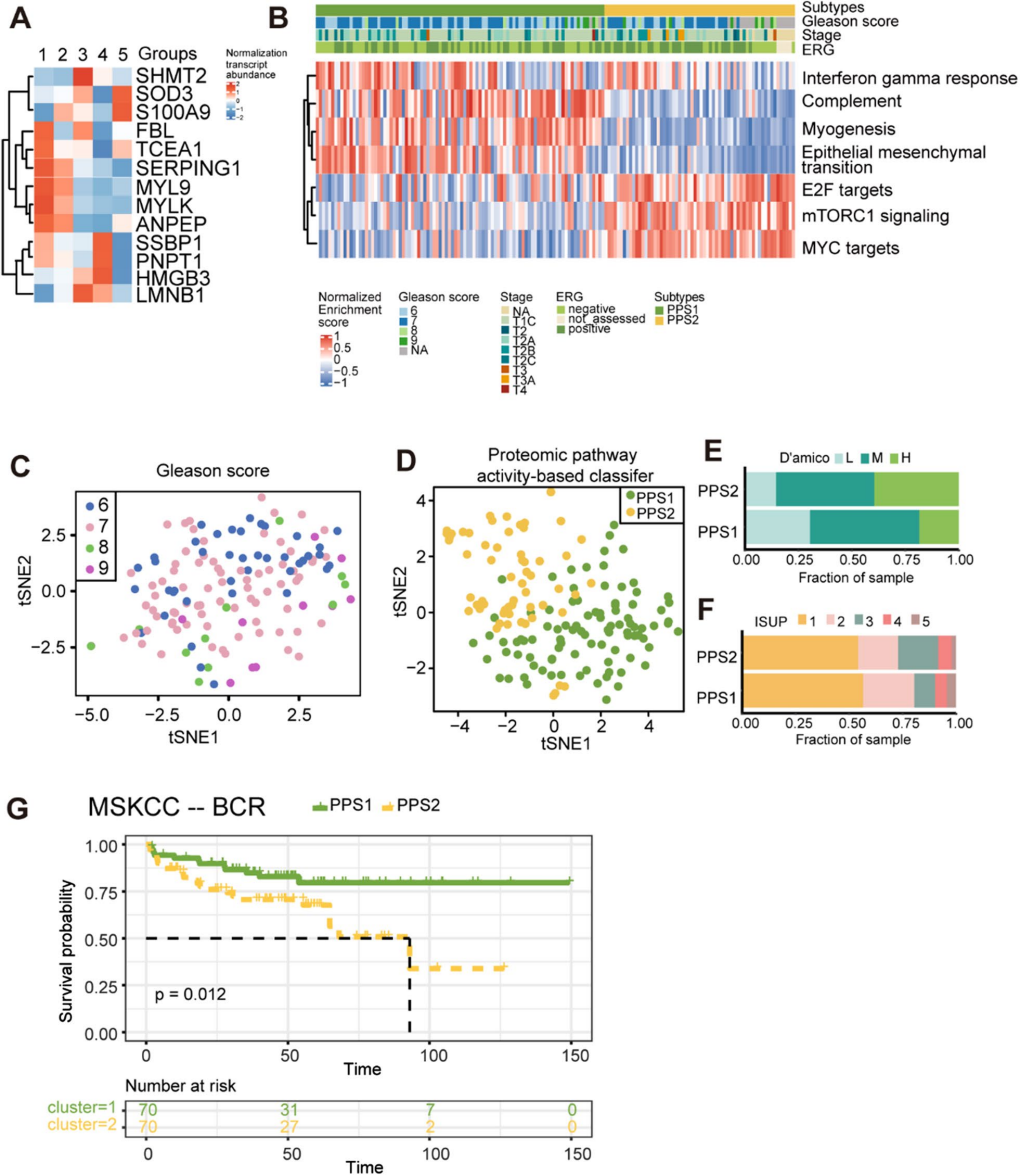
The validation of the proteomic pathways-based classifier in the MSKCC dataset.** A** Heatmap showing the expression of 13 transcripts. The expression of transcript was normalized by Z-score across all PCa patients. **B** Unsupervised classification based on 13 transcripts enriched pathways at the transcriptomic level.** C**–**D** The t-SNE plots show the distribution of all tumor samples based on the ISUP standard and the pathway-based classifier utilizing the selected 13 transcripts, as depicted in Fig. 3A. **E**–**F** Overlay of proteomic-pathway-based subtypes with D’amico (**E**) and ISUP (**F**) classification standard for PCa. **G** Kaplan–Meier curves for the BCR-free survival between the two subtypes

**Fig. 4:**
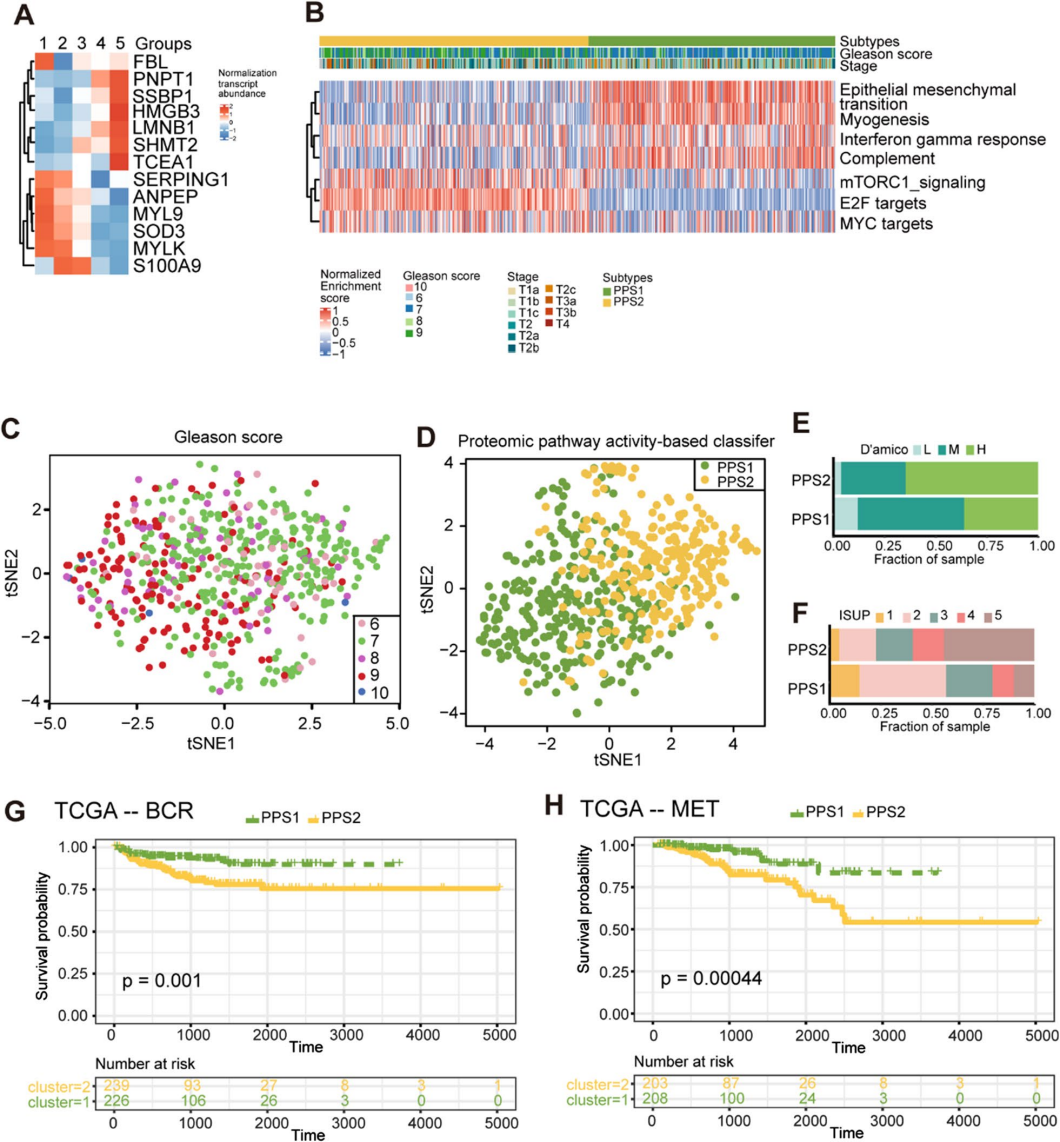
The validation of the proteomic pathways-based classifier in the TCGA dataset.** A** Heatmap showing the expression of 13 transcripts. The expression of transcript was normalized by Z-score across all PCa patients. **B** Unsupervised classification based on 13 transcripts enriched pathways at the transcriptomic level.** C**–**D** The t-SNE plots show the distribution of all tumor samples based on the ISUP standard and the pathway-based classifier utilizing the selected 13 transcripts, as depicted in Fig. 4A. **E**–**F** Overlay of proteomic-pathway-based subtypes with D’amico (**E**) and ISUP (**F**) classification standard for PCa. **G**–**H** Kaplan–Meier curves for the BCR-free (**G**) and metastasis-free (**H**) survival between the two subtypes

To assess whether the seven pathway-based classifier can be used for prognosis prediction, we validated it using two transcriptomic datasets with follow-up records from Western cohorts, one is the MSK-IMPACT clinical sequencing cohort (MSKCC) and the other is from TCGA. For the aforementioned 13 proteins, they were found in both datasets (Fig. 3A for MSKCC, 4A for TCGA). They were all enriched into the same seven pathways. A total of 140 tumor samples (from MSKCC) and 476 tumors (from TCGA) were classified into two subtypes (PPS1 and PPS2) based on the enrichment scores of the seven pathways using the transcriptomic data (Additional file 1: Table S3 C–D, Figs. 3B, 4B). While 13 DEPs were insufficient for differentiating between ISUP grades (Figs. 3C, 4C), our proteomic pathway activity-based classifier was able to effectively categorize PCa patients into two distinct groups (Figs. 3D, 4D). We determined the PCa pathological grades for each sample following the D’Amico [39] and ISUP [2] standards and compared them with our proteomic pathway-based classifier (Figs. 3E, F, 4E, F). PPS1 contained more low-grade PCa patients (ISUP grades 1, 2, 3), and fewer high-grade patients (ISUP grades 4, 5) (Figs. 3E, 4E, F). However, in the MSKCC dataset, patients with higher grades do not have an advantage in terms of proportion in PPS1 compared to PPS2 (Fig. 3F). This may be due to the imbalance of high-grade and low-grade patients in the MSKCC dataset (high vs low = 0.10), which is not as balanced as our proteomic dataset (high vs low = 0.59) and the TCGA dataset (high vs low = 0.68). Further validation in dependent and larger patient cohorts is needed. Innate immune was suppressed and cell proliferation associated pathways were activated in the PPS2 (Figs. 3B, 4B). Interestingly, PPS2 in both datasets had significantly shorter biochemical recurrence (BCR)-free survival than the other two subtypes (log-rank p = 0.012 in MSKCC, Fig. 3G; and log-rank p = 0.001 in TCGA, Fig. 4E). Further, in the TCGA database, PPS2 also showed poor metastasis free survival (Fig. 4H).

Additionally, we compared the mutations and copy number alterations (CNAs) in the seven pathways of the two subtypes using genomic data from TCGA and MSKCC. The highest CNA burden was found in PPS2 in both datasets (Additional file 2: Figure S3A), which exhibited a poorer prognosis. However, the genomic alteration patterns (Additional file 2: Figure S3A) and the main cluster-specific mutated genes varied between the two datasets (Additional file 2: Figure S3B). This finding agrees with our previous finding that high genomic heterogeneity could be buffered at the proteomic level [12].

Altogether, our results demonstrate that PPS2 with the poorest prognosis was characterized by the suppression of innate immunity, which was consistent across multi-omic levels. The seven-pathway based classifier might be used for prognostic prediction in clinics. More validations in prospective clinical trials will be required in the future.

## Conclusions

In summary, this study presents a seven-pathway-based classifier for PCa prognosis prediction. Notably, this classifier may predict BCR/metastasis-free survival and has been validated in two transcriptomic datasets. This study also uncovers dysregulated proteins and pathways associated with PCa progression, which might be a resource for mining novel therapeutic targets for PCa. Pathway-based classification, to some extent, may alleviate the challenges posed by proteins that are not detectable by mass spectrometry in certain samples. Furthermore, the proteomic pathway-based stratification of PCa offers valuable insights into the tumor biology of this cancer.

## Materials and methods

### Peptide sample preparation and pulseDIA analysis

Protein extraction and peptide digestion were performed as the described previously [40]. In brief, about 0.5 mg of FFPE PCa samples were processed to obtain clean peptides through dewaxing, rehydration, protein denaturation, and digestion. The clean peptide samples were separated using the Eksigent NanoLC 400 system. The parameters of the LC system were kept as in a previous study [41]. Peptides were introduced into the TripleTOF 6600 (Sciex) with a DuoSprary source replumbed using 25 µM ID hybrid electrodes to minimize postcolumn dead volume. The mass ranges for acquiring the MS1 and MS2 spectra were 350–1250 *m/z*, and 100–1500 *m/z*, respectively. A 70-variable Q1 isolation window scheme was set, and the accumulation time was set to 20 ms per isolation window.

Before the raw file interpretation, an in-silico DIA-based library was built. Firstly, the raw files were converted to the mzML format using MSConvert. The DIA-NN (1.8.0-Linux version) was then used to construct the DIA-based library using a library-free strategy. Next, the algorithm parameters were set to “unrelated runs” and “match-between-runs (MBR)”. Mass accuracy, MS1 accuracy, and scan window were set to 0 to allow for the automatic optimization by DIA-NN. Trypsin was selected as the digestion enzyme, and missed cleavages were set to 1. Carbamidomethylation was set as a fixed modification, while N-term methylation excision and methionine oxidation were set as variable modifications. The false discovery rates (FDRs) for peptides and proteins were set to 1%. Other parameters were left to their default values, with the exception of “protein inference”, which was set to “protein names” (from FASTA). The background used was a human FASTA file downloaded from the UniProt proteome dataset on January 26th, 2020.

The raw files were then re-searched using our in-silico DIA-based library. The parameters were set as in the above-described step. Next, the peptide files were combined as described in a published report [19]. After filtering out the proteins missing in over 80% of the samples, the remaining 5360 proteins were used in the subsequent analyses. The missing values were imputed by the sequential k-Nearest Neighbor method [42].

### Pathway analysis

The pathway enrichment of the differentially expressed proteins (DEPs) was performed using STRING [43] (Additional file 2: Figure S1A, 2B). The most significantly enriched pathways had a p-value < 0.05 and contained at least two proteins from our dataset.

### Statistical analysis

A two-sided unpaired Welch’s *t*-test was used for the comparison between the two groups. The one-way analysis of variance (ANOVA) was used to determine the difference among different GS grades. P-values were adjusted by the Benjamini & Hochberg method.

### Mfuzz analysis

The average protein quantities in each GS grade were used for fuzzy c-means clustering with the R (version 4.0.2) package Mfuzz (version 2.48.0). The number of clusters was set to ten and the fuzzifier coefficient, M, was set to 1.25.

### Proteomic-based clustering analysis

The enrichment analysis of pathways was performed using the “enricher” function from the “clusterProfiler” package [44] (default parameters) with the utilization of the 50 hallmark gene sets downloaded from MsigDB [45] (Molecular Signature Database v7.4). For the proteomic data, enrichment was conducted using the “gsva” method within the GSVA framework [46]. Similarly, for the transcriptomic data, enrichment was performed using the Pathway Level analysis of Gene Expression method. Each pathway was required to include a minimum of two proteins or transcripts to be considered. The activation score of each pathway was calculated using GSVA, considering the identified proteins or transcripts associated with the respective pathway.

We performed K-means clustering (with the “kmeans” function in R), consensus clustering (the “consensusClusterPlus” package in R), and NbClust testing (the “NbClust” function in R) to determine the optimal number of stable PCa subtypes. We scaled each sample to cluster them based on the constituent pattern of each pathway. Then consensus clustering was used to assess the robustness of the K-means clustering (1000 interactions, 80% resampling). NbClust testing provided 30 different test methods for determining the optimal number of clusters. A silhouette analysis was then performed to confirm the robustness of the clustering.

### Cox regression model

We first excluded samples without survival follow-up data. Then, we randomly divided the data into a training set (80% of the samples) and a test set (20% of the samples). Using the training data, we constructed a Cox model and applied it to predict the risk scores for the test data. Subsequently, based on the median of the risk scores in the test dataset, the samples were divided into high- and low-risk groups. Finally, Kaplan–Meier curves were generated for the high- and low-risk groups in the test dataset.

### Comparison of oncogenic pathway alteration frequencies among subtypes

Seven signaling pathways consisting of 13 genes were evaluated. For each PCa subtype, we computed the fraction of samples with at least one alteration in each of the seven signaling pathways and then compared the two subtypes. A tumor sample was considered pathway-altered if one or more genes from a specific pathway contained a recurrent or known driver alteration.

### Supplementary Information

**Additional file 1: Table S1. **Information of patients and samples. **Table S2.** The differentially expressed analysis of PCa. **Table S3.** The proteomic pathway-based classification for PCa.

**Additional file 2: Figure S1.** Differentially expressed proteins. **A** Pathway enrichment of the dysregulated proteins from comparing tumor and adjacent benign samples (B-H adjusted P-value < 0.05) from STRING [43] (P-value < 0.05). **B** Mfuzz clustering analysis of protein expression across the different groups (One-way ANOVA, B-H adjusted P-value < 0.05). **Figure S2.** Consensus clustering of the proteomic data. The subgroups are identified based on proteomic data by K-means consensus clustering upon their abundance. **Figure S3.** Genomic analysis for the proteomic pathway-based subtypes. **A** The genomic alterations in the seven pathways were compared among the three clusters of proteomic-pathway-based subtypes, including gene mutation frequency (green), amplification frequency (red), and deletion frequency (blue). **B** Sankey diagrams for the mutation frequencies of the genes showing significant P-value (P<0.05) in the comparison between all possible pairs between the two subtypes. The color of the gene name represents the subtype where each gene shows the highest mutation frequency. ANOVA P-value: * <0.05; ** <0.01; *** <0.001.

## Data Availability

The MSK-IMPACT clinical sequencing cohort (MSKCC) were downloaded from cBioPortal [34], while the TCGA data were downloaded from the portal: https://xenabrowser.net/. The MS-based proteomic data have been deposited to the iProX (IPX0003801001).

## References

[1] Sung H, Ferlay J, Siegel RL, Laversanne M, Soerjomataram I, Jemal A (2021). Global cancer statistics 2020: GLOBOCAN estimates of incidence and mortality worldwide for 36 cancers in 185 countries. CA Cancer J Clin.

[2] Epstein JI, Egevad L, Amin MB, Delahunt B, Srigley JR, Humphrey PA, et al. The 2014 International Society of Urological Pathology (ISUP) Consensus Conference on Gleason Grading of Prostatic Carcinoma: Definition of Grading Patterns and Proposal for a New Grading System. Am J Surg Pathol. 2016;40(2):244–52. 10.1097/PAS.0000000000000530 26492179

[3] Erickson A, Sandeman K, Lahdensuo K, Nordling S, Kallajoki M, Seikkula H (2018). New prostate cancer grade grouping system predicts survival after radical prostatectomy. Hum Pathol.

[4] Allsbrook WC, Mangold KA, Johnson MH, Lane RB, Lane CG, Amin MB (2001). Interobserver reproducibility of Gleason grading of prostatic carcinoma: urologic pathologists. Hum Pathol.

[5] Melia J, Moseley R, Ball RY, Griffiths DF, Grigor K, Harnden P (2006). A UK-based investigation of inter- and intra-observer reproducibility of Gleason grading of prostatic biopsies. Histopathology.

[6] Ozkan TA, Eruyar AT, Cebeci OO, Memik O, Ozcan L, Kuskonmaz I (2016). Interobserver variability in Gleason histological grading of prostate cancer. Scand J Urol.

[7] Egevad L, Ahmad AS, Algaba F, Berney DM, Boccon-Gibod L, Comperat E (2013). Standardization of Gleason grading among 337 European pathologists. Histopathology.

[8] Cancer Genome Atlas Research N (2015). The molecular taxonomy of primary prostate cancer. Cell.

[9] Li J, Xu C, Lee HJ, Ren S, Zi X, Zhang Z (2020). A genomic and epigenomic atlas of prostate cancer in Asian populations. Nature.

[10] Stelloo S, Nevedomskaya E, Kim Y, Schuurman K, Valle-Encinas E, Lobo J (2018). Integrative epigenetic taxonomy of primary prostate cancer. Nat Commun.

[11] Fraser M, Sabelnykova VY, Yamaguchi TN, Heisler LE, Livingstone J, Huang V (2017). Genomic hallmarks of localized, non-indolent prostate cancer. Nature.

[12] Charmpi K, Guo T, Zhong Q, Wagner U, Sun R, Toussaint NC (2020). Convergent network effects along the axis of gene expression during prostate cancer progression. Genome Biol.

[13] Sinha A, Huang V, Livingstone J, Wang J, Fox NS, Kurganovs N (2019). The proteogenomic landscape of curable prostate cancer. Cancer Cell.

[14] Gao Q, Zhu H, Dong L, Shi W, Chen R, Song Z (2019). Integrated proteogenomic characterization of hbv-related hepatocellular carcinoma. Cell.

[15] Xu JY, Zhang C, Wang X, Zhai L, Ma Y, Mao Y (2020). Integrative proteomic characterization of human lung adenocarcinoma. Cell.

[16] Jiang Y, Sun A, Zhao Y, Ying W, Sun H, Yang X (2019). Proteomics identifies new therapeutic targets of early-stage hepatocellular carcinoma. Nature.

[17] Gao J, He J, Zhang F, Xiao Q, Cai X, Yi X (2022). Integration of protein context improves protein-based COVID-19 patient stratification. Clin Proteomics.

[18] Guo T, Kouvonen P, Koh CC, Gillet LC, Wolski WE, Rost HL (2015). Rapid mass spectrometric conversion of tissue biopsy samples into permanent quantitative digital proteome maps. Nat Med.

[19] Cai X, Ge W, Yi X, Sun R, Zhu J, Lu C (2021). PulseDIA: data-independent acquisition mass spectrometry using multi-injection pulsed gas-phase fractionation. J Proteome Res.

[20] Knezevic D, Goddard AD, Natraj N, Cherbavaz DB, Clark-Langone KM, Snable J (2013). Analytical validation of the oncotype DX prostate cancer assay—a clinical RT-PCR assay optimized for prostate needle biopsies. BMC Genomics.

[21] Iglesias-Gato D, Wikstrom P, Tyanova S, Lavallee C, Thysell E, Carlsson J (2016). The proteome of primary prostate cancer. Eur Urol.

[22] Kumar L, Matthias EF (2007). Mfuzz: a software package for soft clustering of microarray data. Bioinformation.

[23] Chakravarthi B, Chandrashekar DS, Agarwal S, Balasubramanya SAH, Pathi SS, Goswami MT (2018). miR-34a regulates expression of the stathmin-1 oncoprotein and prostate cancer progression. Mol Cancer Res.

[24] Yamada Y, Nishikawa R, Kato M, Okato A, Arai T, Kojima S (2018). Regulation of HMGB3 by antitumor miR-205-5p inhibits cancer cell aggressiveness and is involved in prostate cancer pathogenesis. J Hum Genet.

[25] Koh CM, Gurel B, Sutcliffe S, Aryee MJ, Schultz D, Iwata T (2011). Alterations in nucleolar structure and gene expression programs in prostatic neoplasia are driven by the MYC oncogene. Am J Pathol.

[26] Matsunaga S, Takata H, Morimoto A, Hayashihara K, Higashi T, Akatsuchi K (2012). RBMX: a regulator for maintenance and centromeric protection of sister chromatid cohesion. Cell Rep.

[27] Marrocco I, Altieri F, Rubini E, Paglia G, Chichiarelli S, Giamogante F (2019). Shmt2: a Stat3 signaling new player in prostate cancer energy metabolism. Cells.

[28] Kim J, Mizokami A, Shin M, Izumi K, Konaka H, Kadono Y (2014). SOD3 acts as a tumor suppressor in PC-3 prostate cancer cells via hydrogen peroxide accumulation. Anticancer Res.

[29] Andor N, Graham TA, Jansen M, Xia LC, Aktipis CA, Petritsch C (2016). Pan-cancer analysis of the extent and consequences of intratumor heterogeneity. Nat Med.

[30] Takahashi S, Suzuki S, Inaguma S, Ikeda Y, Cho YM, Hayashi N (2003). Down-regulated expression of prostasin in high-grade or hormone-refractory human prostate cancers. Prostate.

[31] Minner S, Hager D, Steurer S, Hoflmayer D, Tsourlakis MC, Moller-Koop C (2019). Down-regulation of S100A8 is an independent predictor of PSA recurrence in prostate cancer treated by radical prostatectomy. Neoplasia.

[32] Wang JH, Zhang L, Huang ST, Xu J, Zhou Y, Yu XJ (2017). Expression and prognostic significance of MYL9 in esophageal squamous cell carcinoma. PLoS ONE.

[33] Davis JN, Wojno KJ, Daignault S, Hofer MD, Kuefer R, Rubin MA (2006). Elevated E2F1 inhibits transcription of the androgen receptor in metastatic hormone-resistant prostate cancer. Cancer Res.

[34] Taylor BS, Schultz N, Hieronymus H, Gopalan A, Xiao Y, Carver BS (2010). Integrative genomic profiling of human prostate cancer. Cancer Cell.

[35] Latonen L, Afyounian E, Jylha A, Nattinen J, Aapola U, Annala M (2018). Integrative proteomics in prostate cancer uncovers robustness against genomic and transcriptomic aberrations during disease progression. Nat Commun.

[36] Meng J, Lu X, Jin C, Zhou Y, Ge Q, Zhou J (2021). Integrated multi-omics data reveals the molecular subtypes and guides the androgen receptor signalling inhibitor treatment of prostate cancer. Clin Transl Med.

[37] Lapointe J, Li C, Giacomini CP, Salari K, Huang S, Wang P (2007). Genomic profiling reveals alternative genetic pathways of prostate tumorigenesis. Cancer Res.

[38] Rebello RJ, Pearson RB, Hannan RD, Furic L (2017). Therapeutic approaches targeting MYC-driven prostate cancer. Genes.

[39] D'Amico AV, Whittington R, Malkowicz SB, Schultz D, Blank K, Broderick GA (1998). Biochemical outcome after radical prostatectomy, external beam radiation therapy, or interstitial radiation therapy for clinically localized prostate cancer. JAMA.

[40] Zhu Y, Weiss T, Zhang Q, Sun R, Wang B, Yi X (2019). High-throughput proteomic analysis of FFPE tissue samples facilitates tumor stratification. Mol Oncol.

[41] Sun R, Hunter C, Chen C, Ge W, Morrice N, Liang S (2020). Accelerated protein biomarker discovery from FFPE tissue samples using single-shot, short gradient microflow SWATH MS. J Proteome Res.

[42] Kim KY, Kim BJ, Yi GS (2004). Reuse of imputed data in microarray analysis increases imputation efficiency. BMC Bioinformatics.

[43] Szklarczyk D, Gable AL, Nastou KC, Lyon D, Kirsch R, Pyysalo S (2021). The STRING database in 2021: customizable protein-protein networks, and functional characterization of user-uploaded gene/measurement sets. Nucleic Acids Res.

[44] Yu G, Wang LG, Han Y, He QY (2012). clusterProfiler: an R package for comparing biological themes among gene clusters. OMICS.

[45] Liberzon A, Birger C, Thorvaldsdottir H, Ghandi M, Mesirov JP, Tamayo P (2015). The molecular signatures database (MSigDB) hallmark gene set collection. Cell Syst.

[46] Hanzelmann S, Castelo R, Guinney J (2013). GSVA: gene set variation analysis for microarray and RNA-seq data. BMC Bioinf.

