# Supplementary material for: A pathway activity-based proteomic classifier stratifies prostate tumors into two subtypes
Source: Clin Proteomics. 2023 Nov 11;20:50. doi: 10.1186/s12014-023-09441-w (PMC10638831; doi:10.1186/s12014-023-09441-w)
Supplement: Supplementary file 2 — Additional file 2: Figure S1. Differentially expressed proteins. A Pathway enrichment of the dysregulated proteins from comparing tumor and adjacent benign samples (B-H adjusted P-value < 0.05) from STRING [43] (P-value < 0.05). B Mfuzz clustering analysis of protein expression across the different groups (One-way ANOVA, B-H adjusted P-value < 0.05). Figure S2. Consensus clustering of the proteomic data. The subgroups are identified based on proteomic data by K-means consensus clustering upon their abundance. Figure S3. Genomic analysis for the proteomic pathway-based subtypes. A The genomic alterations in the seven pathways were compared among the three clusters of proteomic-pathway-based subtypes, including gene mutation frequency (green), amplification frequency (red), and deletion frequency (blue). B Sankey diagrams for the mutation frequencies of the genes showing significant P-value (P<0.05) in the comparison between all possible pairs between the two subtypes. The color of the gene name represents the subtype where each gene shows the highest mutation frequency. ANOVA P-value: * <0.05; ** <0.01; *** <0.001. [file 12014_2023_9441_MOESM2_ESM.docx]

Supplementary Figure 1


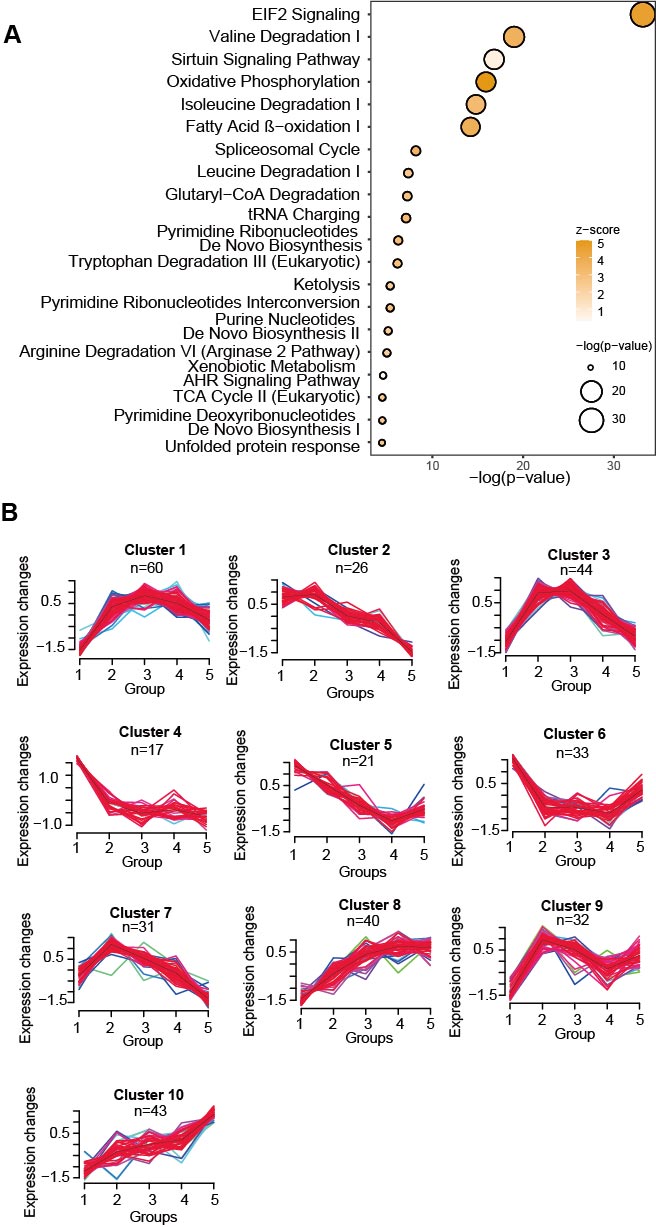


**Figure S1.** Differentially expressed proteins. **(A)** Pathway enrichment of the dysregulated proteins from comparing tumor and adjacent benign samples (B-H adjusted P-value < 0.05) from STRING(43) (P-value < 0.05). **(B)** Mfuzz clustering analysis of protein expression across the different groups (One-way ANOVA, B-H adjusted P-value < 0.05).

Supplementary Figure 2


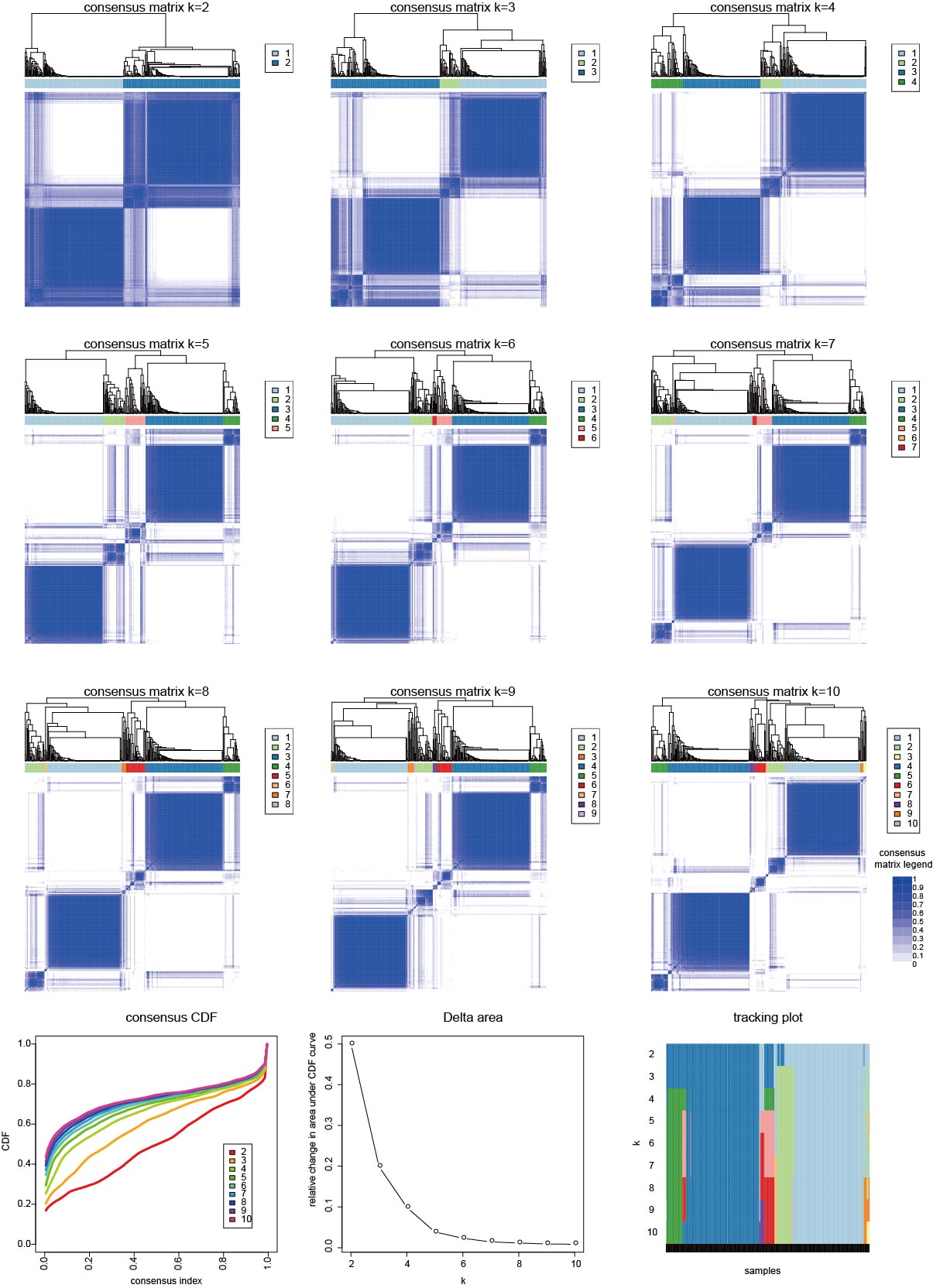


**Figure S2.** Consensus clustering of the proteomic data. The subgroups are identified based on proteomic data by K-means consensus clustering upon their abundance.

Supplementary Figure 3


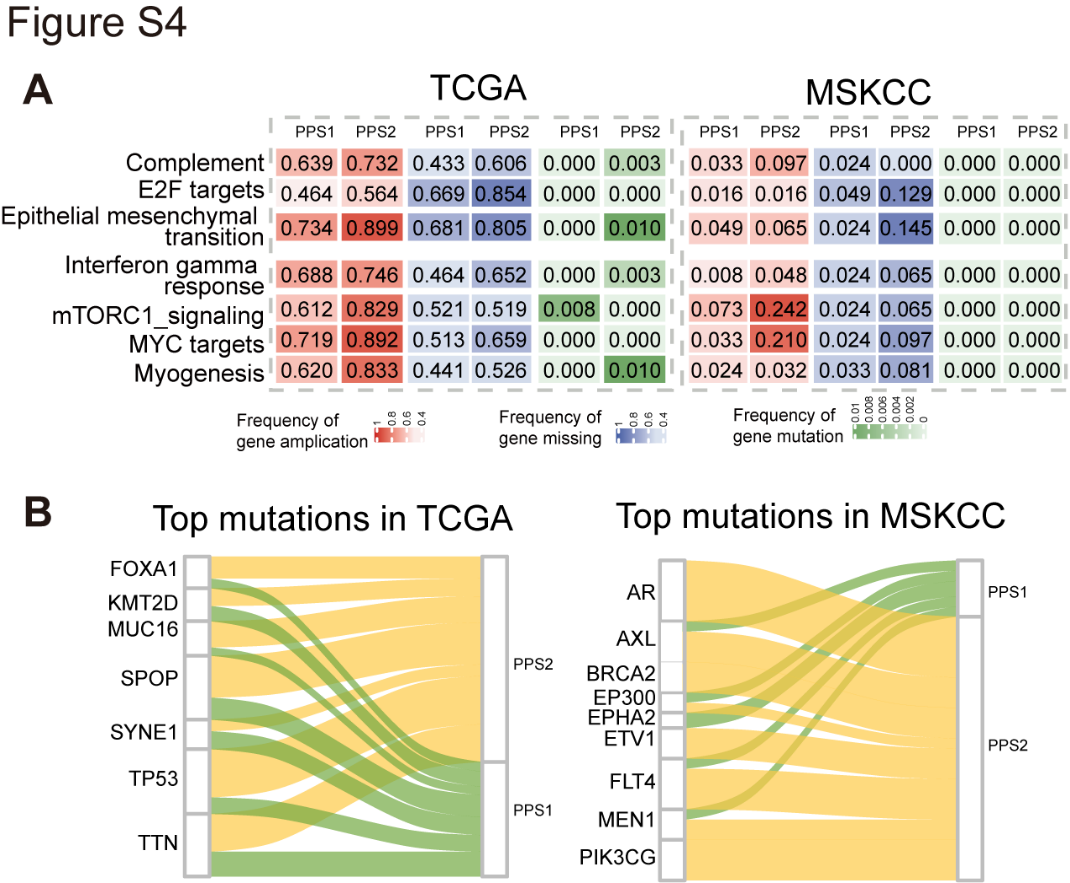


**Figure S3.** Genomic analysis for the proteomic pathway-based subtypes. **(A)** The genomic alterations in the seven pathways were compared among the three clusters of proteomic-pathway-based subtypes, including gene mutation frequency (green), amplification frequency (red), and deletion frequency (blue). **(B)** Sankey diagrams for the mutation frequencies of the genes showing significant P-value (P<0.05) in the comparison between all possible pairs between the two subtypes. The color of the gene name represents the subtype where each gene shows the highest mutation frequency. ANOVA P-value: * <0.05; ** <0.01; *** <0.001.
